# Histopathology of long head of biceps tendon removed during tenodesis demonstrates degenerative histopathology and not inflammatory changes

**DOI:** 10.1186/s12891-022-05124-z

**Published:** 2022-02-26

**Authors:** Maciej J. K. Simon, Jane Yeoh, Jennifer Nevin, Michael Nimmo, William D. Regan

**Affiliations:** 1 Department of Orthopaedics, Chan Gunn Pavilion, Allen McGavin Sports Medicine Clinic, University of British Columbia, 2553 Wesbrook Mall, Vancouver, BC V6T1Z3 Canada; 2 Department of Orthopaedics and Trauma Surgery, University Medical Center Schleswig-Holstein - Campus Kiel, Arnold-Heller-Str. 3, 24105 Kiel, Germany; 3 Department of Orthopaedics, University of British Columbia, 2775 Laurel Street, 11th Floor, Vancouver, BC V5Z1M9 Canada; 4 Department of Pathology, University of British Columbia, 910 West 10th Avenue, Room 135C, Vancouver, BC V5Z4E3 Canada

**Keywords:** Long head of biceps tendon, Tendinopathy, Tendinosis, Shoulder histology, Histopathology, Outcomes

## Abstract

**Background:**

The aim of this study is to describe and quantitatively analyze the histopathology of proximal long head biceps (LHB) tendinopathy in patients who have undergone LHB tenodesis. The hypothesis is that severe histopathologic changes of the LHB tendon (LHBT) will most likely be reflected with improved postoperative clinical outcomes.

**Methods:**

The study included patients with isolated LHB tendinopathy or LHB tendinopathy associated with concomitant shoulder pathologies. All had failed conservative treatment (12 months) and had a positive pain response (> 50% reduction) pre-operatively after LHB tendon injection with local anesthetic. All underwent biceps tenodesis procedure between 2008 and 2014. Tendon specimens were collected and histologically analyzed with the semi-quantitative Bonar scoring system. Minimum follow-up time was 1 year. A subset of patients was retrospectively reviewed postoperatively and evaluated employing visual analogue score (VAS), short form survey (SF-12), American Shoulder and Elbow Surgeon (ASES) score, Disability of Arm, Shoulder and Hand (DASH) score, and Oxford Shoulder Score (OSS) and postoperative return to work status.

**Results:**

Forty-five biceps tendon specimens were obtained from 44 patients (mean age 50 ± 9.6 years). Histopathological analyses demonstrated advanced degenerative changes with myxoid degeneration and marked collagen disorganization. Minimal inflammation was identified. There were no regional differences in histopathological changes. Clinical outcomes did not correlate significantly with severity of histopathologic changes.

**Conclusions:**

This study confirms that LHBT specimens in patients undergoing tenodesis demonstrate with the use of the Bonar score histopathologic changes of chronic degeneration and not inflammation. The correct histopathologic terminology for this process is LHB tendinosis. The histopathological changes appear uniform throughout the entire length of the LHBT which may inform the nature of the procedure performed.

## Background

Tendinopathy of the long head of the biceps brachii tendon (LHBT) is a known source of anterior shoulder pain [1, 2]. It is commonly associated with rotator cuff and labral tears, acromioclavicular joint pathology and glenohumeral osteoarthritis [3–6].

Theories cited to explain LHB tendinopathy, include hypovascularity, inflammation and repetitive microtrauma from traction, friction and glenohumeral rotation. One or a combination of these factors likely contribute to pathogenesis [7].

Studies of histopathological changes in LHB tendinopathy have identified a variety of alterations including chronic inflammation, fibrosis, myxoid degeneration, collagen disorganization, altered vascularity, and morphologic changes of the tenocytes [8–12]. There is no consensus regarding whether pain emanating from the long head biceps represents a uniform tendinitis or a tendinosis nor if changes within the tendon are clustered regionally. These considerations have been cited by Glait et al. and Nuelle et al. as important in determining the most appropriate site of tenodesis [13].

The goal of this study is to provide a descriptive and quantitative analysis of the histopathology of proximal LHB tendinopathy in patients who have undergone LHB tenodesis. The hypothesis of this study is that the severity of histopathologic changes (particularly inflammatory characteristics) of the LHBT will be reflected with improved postoperative clinical and functional outcomes post tenodesis.

## Methods

This is a retrospective case series of patients who underwent elective LHB tenodesis by the primary surgeon (WR) and histologic assessment of resected LHBT specimens between 2008 and 2014. Patients were retrospectively identified using billing codes for LHB tenodesis.

**Fig. 1:**
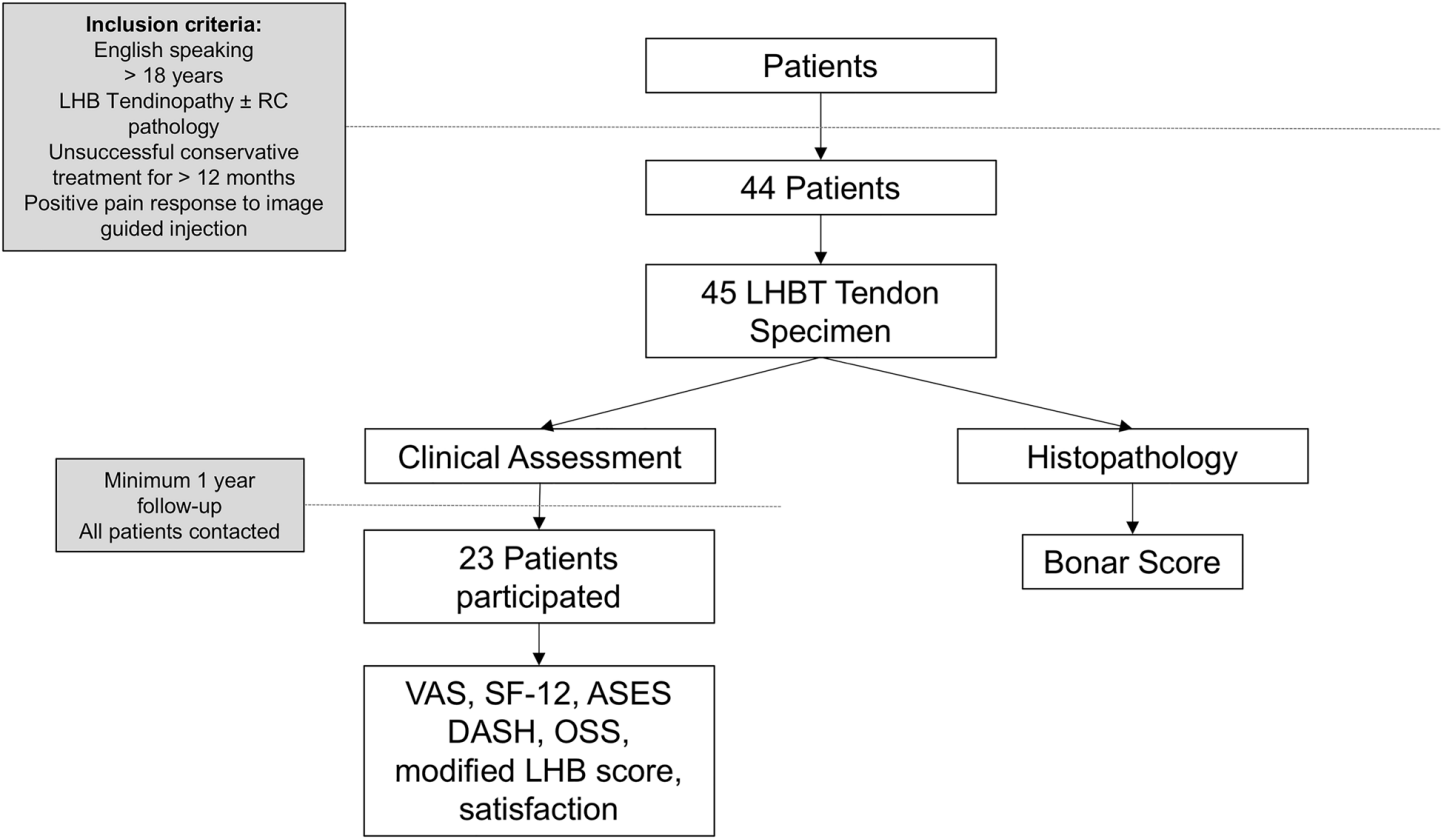
Flowchart demonstrating with inclusion criteria 44 patients could be included in the study. Forty-five specimen of long head biceps tendon (LHBT) were collected for histopathology and 23 patients could be included in a minimum of 1-year follow-up assessment. [VAS: visual analogue scale, SF-12: short form-12, ASES: American Shoulder and Elbow Score, DASH: Disability of the Arm, Shoulder and Hand, OSS: Oxford Shoulder Score, and modified LHB score; RC: Rotator cuff]

Inclusion criteria: English speaking patients greater than 18 y. o. at time of surgery, with isolated LHB tendinopathy or LHB tendinopathy associated with rotator cuff pathology, impingement syndrome or the acromioclavicular joint, who had failed conservative care for > 12 months (Fig. 1). All patients had a positive pain response pre-operatively following (ultrasound guided) injection within the LHBT sheath. Exclusion criteria included non-English speaking patients, or patients with concurrent significant shoulder osteoarthritis, inflammatory arthritides, proximal humerus fracture, or other significant systemic disease.

All investigations were approved by University of British Columbia research ethics prior to collecting data (CREB Approval H13–03258) and performed after obtaining informed consent in accordance with the latest version of the Declaration of Helsinki.

### Patients

All patients presented with anterior shoulder pain and dysfunction. History, physical examination and investigations (MR imaging, MR arthrogram, or ultrasound) confirmed LHB tendinopathy alone or in combination with rotator cuff, or labral pathology. After failing conservative management (12 months) including a positive pain response (> 50% pain reduction) after an ultrasound guided diagnostic LHB injection extra-articularly (5 mg / mL ropivacaine (Ropivacaine Hydrochloride Injection, Fresenius Kabi Canada, Toronto, Canada) plus sometimes a corticosteroid 40 mg / mL (^Pr^DEPO-MEDROL®, Pfizer Canada, Quebec, Canada) for potential therapeutic purposes). All patients underwent either arthroscopic suprapectoral or arthroscopic-assisted subpectoral LHB tenodesis to manage refractory anterior shoulder pain. The patients’ comorbid shoulder pathology (e.g. subacromial decompression labral debridement/repair, rotator cuff reconstruction) was also addressed.

### Surgical procedure and specimen harvest

Shoulder diagnostic arthroscopy was performed in all cases. All proximal LHBT were resected arthroscopically with cutting instruments at its origin as proximal to the biceps anchor as possible. The suprapectoral technique involved tenodesis of the LHBT arthroscopically at the bicipital groove with an interference screw or anchor [14, 15]. In these cases, the tendon was resected arthroscopically following tenodesis at suprapectoral level maintaining biceps tension. The subpectoral tenodesis involved a mini-open subpectoral location technique as described in the literature [16]. Here the proximal portion of LHBT was resected below the pectoral insertion level while maintaining biceps tension. These specimens yielded a different tendon specimen length compared to suprapectoral technique.

All resected LHBT were preserved in formalin (10% Neutral Buffered Formalin, Richard-Allan Scientific LLC, Kalamazoo, Michigan, USA), and sent to pathology for analysis. A cadaveric biceps tendon was obtained, from a 65-year-old male with no reported issues related to the biceps, for comparison. It was stained identically to the pathological tendons.

### Pathological analyses

LHBT specimens were divided into proximal, central and distal segments. The segments were divided by percentage length of the harvested specimen. Each specimen were stained with the routine stain Haematoxylin and Eosin (H&E). In addition, specimens were stained with Trichrome to highlight fibrosis and with Alcian Blue (pH 2.5) to highlight myxoid degeneration.

**Fig. 2:**
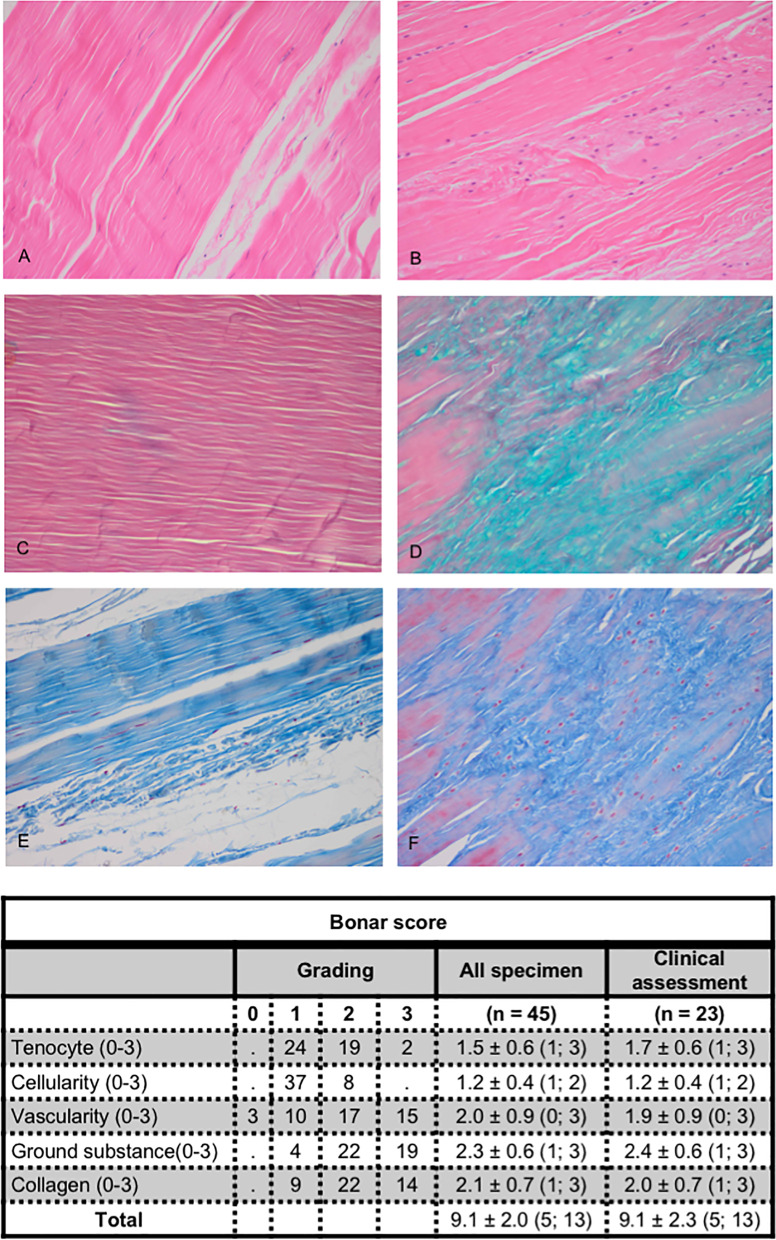
Histopathological images and assessment of the Bonar score. Figures on the left demonstrate a healthy long head biceps tendon (LHBT) and on the right a diseased LHBT. **A** Normal Tendon (H&E × 200); **B** Increased tenocyte cellularity (H&E × 200); **C** Normal Tendon (Alcian blue × 200); **D** Increased ground substance/mucin (staining green) deposition (Alcian blue × 200); **E** Normal Tendon (Tri-chrome × 200); **F** Disorganization and loss of normal parallel arrangement of collagen fibers (Tri-chrome × 200)

The validated Bonar score was tabulated and applied to the tendon sections as previously described [17]. The specimens were evaluated by one senior pathologists on 5 parameters each graded from 0 to 3: (A) Change in tenocyte morphology, (B) Increase in tenocyte cellularity, (C) Increase in vascularity, (D) Deposition of ground substance, (E) Disruption of collagen fiber arrangement. Each parameter was scored as absent (0), mild (1), moderate (2), or severe (3) (Fig. 2). Total score was graded: minimum 0 = no pathological changes to maximum 15 = worst pathological changes. In addition to the Bonar score, tendons were assessed for the presence of inflammation (assessed as present or absent). Healthy and pathological tendons for each histopathological parameter of the Bonar score are demonstrated in Fig. 2*.* Normal tendon on the left (Fig. 2A, C and E) and abnormal tendons on the right showing increased tenocytes (Fig. 2B), increased ground substance deposition (Fig. 2D) and disorganization/disarray of collagen (Fig. 2F).

### Clinical follow-up

All patients were contacted a minimum one-year following surgery and if agreed to participate were clinically assessed by an independent orthopedic surgeon (JY) not involved in their care.

Demographic data collected included age, gender, comorbidities, side of surgery, employment or physical demands, and worker’s compensation (WC)-related injury (Table 1). Clinical and functional outcomes collected included a pain visual analogue scale (VAS), short form survey (SF-12) [18], American Shoulder and Elbow Score (ASES) [19], Disability of the Arm, Shoulder and Hand (DASH) [20], Oxford Shoulder Score (OSS) [21], and modified LHB score [22]. Satisfaction was rated as dissatisfied, neutral or satisfied. In addition, concomitant shoulder diagnoses and postoperative return to work status were tabulated (Table 2). Histological scores were grouped into above and below the mean for comparison (Bonar score ≤ 9 or ≥ 10).

**Table 1:** Patient demographics and preoperative data, all cases (^§^*n* = 45 but 44 patients) and *n* = 23 with clinical follow-up

	***n*** = 45^**§**^	***n*** = 23
**Sex**		
Female	18 (40%)	12 (52.2%)
Male	27 (60%)	11 (47.8%)
**Mean age (± SD, min; max)**	48.8 ± 9.9 (30; 70)	50.9 ± 9.6 (32; 70)
**Average comorbidities (*n*** **= 23)**	1.0 ± 1.1 (0; 4)	1.0 ± 1.1 (0; 4)
**Handedness**		
Right	40 (88.9%)	22 (95.6%)
Left	5 (11.1%)	1 (4.4%)
**Surgical side**		
Right	26 (57.8%)	14 (61%)
Left	19 (42.2%)	9 (39%)
**Employment**		
Employed	39 (86.7%)	19 (82.6%)
Unemployed / Retired	6 (13.3%)	4 (17.4%)
**Workers’ compensation (WC) status**		
WC-related	30 (66.7%)	13 (56.5%)
Non-WC-related	15 (33.3%)	10 (43.5%)

**Table 2:** Perioperative Data. All cases (^a^*n* = 45 but 44 patients) and n = 23 with clinical follow-up

	***n*** = 45^**a**^	***n*** = 23
**LHBT tenodesis site**		
Supra-pectoral	32 (71.1%)	19 (82.6%)
Sub-pectoral	13 (28.9%)	4 (17.4%)
**Concomitant surgical procedures**^b^		
Subacromial decompression	25	11
Rotator cuff repair	11	8
Excision of distal clavicle	2	1
Glenohumeral debridement	14	6
Removal of intratendinous calcification (not LHBT)	1	1
Capsular release	3	0
**Issues with tenodesis site**^c^	**14 (31.1%)**	**7 (30.4%)**
Supra-pectoral	11	5 (61%)
Sub-pectoral	3	2 (39%)
**Non-operative Treatment**	**7 of 14**	**3 of 7**
Pain-medication and physiotherapy only	2	1
Needed Image guided (USS) pain ablation	5	2
**Surgical Revision**	**7 of 14**	**4 of 7**
From proximal to distal supra-pectoral level with anchor fixation	2	2
From supra-pectoral level to sub-pectoral level with anchor fixation	3	1
Staying at sub-pectoral level with anchor fixation	1	0
Staying at sub-pectoral level with all-suture anchor tenodesis	1	1
**Return to work (months)** (*n* = 19)	8.5 ± 10.7 (0; 34)	8.5 ± 10.7 (0; 34)
**Postoperative Follow-up (months)**	21.9 ± 14.2 (12; 55)	34.0 ± 11.1 (12; 55)

^b^multiple concomitant surgical procedures possible per case; ^c^ pain, cramping, tendon rupture, fixation failure, *LHBT* Long head of biceps tendon, *USS* Ultrasound scan

### Statistical Analyses

Histology variables included Bonar score, expressed as continuous totals, binary classifications (above/below average). Outcomes included SF12-PCS, SF12-MCS, DASH, ASES-PVAS, ASES score, OSS and LHB score. Descriptive statistics were computed for all variables. A post-hoc power calculation was performed. Spearman rank correlations were computed (with *p*-values and confidence intervals) between outcomes and histology variables. Linear regression models were fit on outcomes vs. histology variables, adjusted for age, sex and WC-status. Model fit was assessed via normal quantile-quantile plots of the residuals. All analyses were performed using SAS v9.4 (SAS Institute, Cary, North Carolina, USA).

## Results

### Demographics

Forty-five histological specimens from 44 patients were included. Twenty-three patients with 23 specimens agreed to participate in clinical and functional assessment (Fig. 1). The mean age was 50 ± 9.6 years. Of the 23 patients, nineteen participants (82.6%) were employed at the time of surgery, while 4 participants (17.4%) were retired or unemployed. Of the 23 patients, there was an average of 1.0 ± 1.1 comorbidities; 9 of 23 (39%) participants reported no comorbidities (Table 1).

Overall, thirty-two cases underwent suprapectoral tenodesis and thirteen sub-pectoral tenodesis (Table 2). There were no intra-operative complications. No infections or neurologic complications were registered post-operatively. There were 14 patients with postoperative issues (e.g. insertional tenodesis site pain, biceps cramping, rupture/failure of tenodesis). The majority (11 of 14) occurred following suprapectoral tenodesis. Five resolved with corticosteroid infiltration at tenodesis site, the localized pain subsided in two, and seven (5 suprapectoral, 2 subpectoral) required revision. Two were revised to a distal suprapectoral level, three were revised from suprapectoral level to subpectoral and one was revised with anchor fixation at previous subpectoral level. The final case with painful fixation at subpectoral level was revised by hardware removal and utilization of an all-suture anchor tenodesis at subpectoral level. Comorbid surgeries are tabulated in Table 2.

### Histology and Bonar score

Of the total 45 LHBT samples, the mean Bonar score was 9.1 ± 2.0 (range 5 to 13) (Fig. 2). Of 23 participants who returned for clinical FU, the average Bonar score was 9.1 ± 2.3 (range 5 to 13) (Fig. 2).

Overall, myxoid degeneration (ground substance) and collagen changes achieved the highest grading levels demonstrating chronic degenerative changes, 2.3 ± 0.6 and 2.1 ± 0.7 respectively (Fig. 2). Analysis demonstrated inflammation in a minority of cases (no inflammation = 33 cases and inflammation present = 12 cases). The inflammation identified consisted of chronic inflammatory cells and was minimal. H&E stained sections from each segment of the tendons (proximal, mid, distal) were also assessed to determine whether there were regional differences in histopathological changes adjusting for tendon length according to suprapectoral and subpectoral tenodesis. However, no regional differences were identified. Most cases demonstrated uniform degenerative changes involving the entire length of the tendon. Finally, there was no significant difference in histological score between participants who completed clinical evaluation and those who did not (*p* = 0.40).

### LHB tendon histology score and postoperative clinical outcomes

Neither Bonar score nor inflammation were statistically associated with postoperative outcome scores (Table 3). The postoperative improvement in the SF-12 MCS, DASH and ASES were superior for the high grade (≤10) Bonar group when compared with the low-grade group. However, there were no statistical differences (*p* > 0.213) (Table 3).

**Table 3:** LHB Tendon (LHBT) Histology Score and Postoperative Clinical Outcomes

	Total / All	Low grade BONAR score (≤9)	High grade BONAR score (≥10)	***P***-value
**Patients (n)**	23	14 (60.9%)	9 (39.1%)	
**VAS (0–10)**	2.2 ± 2.4 (0; 7)	2.1 ± 2.2 (0; 7)	2.4 ± 2.7 (0; 7)	0.536
**SF-12 PCS (0–100, 100 best)**	45.6 ± 9.0 (30.9; 56.8)	46.3 ± 8.4 (30.9;56.8)	44.6 ± 10.3 (32.3; 56.8)	0.807
**SF-12 MCS (0–100, 100 best)**	52.7 ± 11.5 (24; 66.4)	50.6 ± 13.0 (24; 61.9)	55.9 ± 8.5 (41.1; 66.4)	0.234
**DASH (0–100, 100 worst)**	21.5 ± 22.0 (0; 85)	23.9 ± 25.3 (0; 85)	17.6 ± 16.1 (0; 40)	0.213
**OSS (12–60, 60 worst)**	29.8 ± 10.4 (15; 49)	29.2 ± 10.8 (16; 49)	30.8 ± 10.3 (15; 48)	0.860
**Patients (n)**	21	14 (66.7%)	7 (33.3′%)	
**ASES (0–100, 100 best)**	75.9 ± 21.7 (21.7; 100)	73.6 ± 22.7 (21.7; 100)	80.6 ± 20.2 (51.7; 100)	0.229
**Patients (n)**	17	11 (64.7%)	6 (35.3%)	
**LHB Score (0–100, 100 best)**	73.7 ± 16.7 (38; 94)	76.0 ± 15.9 (40; 94)	69.6 ± 18.7 (38; 85)	0.712

*VAS* Visual analogue scale, *SF-12* Short form-12, *ASES* American Shoulder and Elbow Score, *DASH* Disability of the Arm, Shoulder and Hand, *OSS* Oxford Shoulder Score, and modified LHB score

### Return to work status

Of 23 patients, 19 patients (11 WC-status) were employed prior to surgery. Four were retired or unemployed. Postoperatively, seven (36.8%) (3 WC-status) returned to work at their pre-surgery status. Another seven (36.8%) (5 WC-status) returned to work, but to modified duties due to persistent shoulder symptoms. Four (21.1%) (2 WC-status) did not return to work due to their shoulder symptoms. These patients had all been off work > 1 year preoperative. One (0.5%) (1 WC-status) did not return to work for reasons other than shoulder symptoms.

### Satisfaction

Of 23 patients, 15 (65%) were satisfied they had shoulder surgery (8 WC-related and 7 non-WC-related patients), two (8.7%) were neutral about the outcome of their shoulder surgery (1 WC-related and 1 non-WC-related patients), and six (26%) were dissatisfied with their shoulder surgery at final follow-up (4 WC-related and 2 non-WC-related patients).

## Discussion

The histopathological analyses of LHBT post tenodesis did not show any signs of an acute inflammatory process. Inflammation was identified in a minority of cases and consisted of mild, focal, chronic inflammation. The hypothesis that the severity of histopathological tendon changes associate with improved postoperative clinical outcomes was not supported. The major histopathologic changes were collagen disorganization and myxoid degeneration consistent with a tendinosis, not a tendinitis.

Recently, Nuelle et al. analyzed histologically the resected LHBT for the presence of regional histological changes following the separation of the tendon into 3 parts (proximal, middle and distal) [13]. The authors identified that the two proximal parts had significantly higher Bonar scores than the distal and postulated that this was secondary to regional tenosynovitis, a localized inflammatory site and pain source [13]. Unfortunately, the correlation for all three parts was weak (proximal *r* = 0.08; middle *r* = 0.03; distal *r* = 0.1) and there was no apparent inflammation but rather synovial hypertrophy and hyperplasia.

Glait and colleagues examined the different LHB tendon sections but also looked at clinical outcomes employing DASH, ASES and VAS [23]. They compared outcomes of patients with comorbid superior labral anterior posterior (SLAP) lesions versus rotator cuff (RC) lesions. Their analyses did not identify significant differences in outcomes between comorbid SLAP or RC cases, but did identify significantly more mucinous degeneration in the proximal and partially in the middle sections of the tendon specimens taken from patients undergoing a concomitant rotator cuff repair.

The current study confirms the major histopathologic findings of both former studies which include mucinious/myxoid material deposition and fibrotic (collagen) changes as the most commonly seen pathologic changes in the tendons analyzed and that inflammation was minimal. Similar results were described by Zabrzyński et al. who examined 35 LHBT samples and identified a significant disorganization of the collagen bundles, scattered tenocytes and significant degeneration of ground substance [24]. Furthermore, in most cases (32 out 35) degenerative processes of the tendon were identified without any signs of inflammation, despite the fact that 94% of these cases had a positive clinical test for a biceps pathology. The current study did not identify any appreciable histological differences between the three segmental regions of the LHBT. The authors noted instead relatively uniform morphological changes throughout the tendon. The histomorphological features overall confirm that this is not an inflammatory process but rather a uniform tendinosis [9, 12, 25–27]. Our study confirms findings of others who describe chronic tendon degeneration and a paucity of inflammation [9, 25, 28, 29]. Inflammation may play an important role immediately following tendon damage. It appears that after a year of conservative treatment, inflammation plays a minor, if any, role in the symptomatology.

Clinical outcome scores were not associated with histological severity, hence routine specimen collection of a non-suspiciously/non-malignant looking LHBT for histopathological analysis taken during the process of tenodesis is not recommended. The current study identified on average only moderate histopathological changes, uniformly distributed according to the Bonar score (9.1 ± 2.3) despite the analysis of the entire LHBT. Fearon et al. suggest to use the area of the tendon with the worst histopathological Bonar score in order to identify the worst degree of tendinopathy and use this section’s score for representative purposes despite that, we and similarly, Zabrzynski et al. did not identify any correlation of the histopathological changes of the LHBT by the Bonar score and clinical outcomes [30]. The LHBT is a unique tendon as its main portion is located intraarticularly and therefore the tendon histology can be different to other, particularly extraarticular, tendons of the body. Thus, this may lead to altered histopathology and clinical manifestations.

Patient satisfaction was noted to be inferior in cases where the LHBT was tenodesed in the suprapectoral location. The current authors recognize other authors who reported that proximal LHB tenodesis fixation may result in residual pain due to tenosynovitis within the biceps sheath or tendinosis of distal tendon [31–33]. In fact, the current authors noted increased postoperative pain issues in suprapectoral tenodesis cases (11 out 14 cases) which required further treatment (injections and/or revision surgery). The previous studies and our results confirm that distal fixation of the LHBT (subpectoral) is preferred to reduce postoperative complications [31, 32, 34]. This is in keeping with our analysis of histopathology which demonstrates a uniform tendinosis. Other issues to be included in the discussion are the fixation methods of the LHBT. There are cadaver studies examining the onlay versus an inlay technique of the LHB tenodesis with the use of a suture anchor fixation at the superior aspect of the bicipital groove [35]. However, their aim was load to failure, which was in favor of the inlay group with 215 N versus 210 N for the onlay technique. Another cadaver study by Tashjian et al. compared different subpectoral fixation methods of the LHB tenodesis [36]. They analyzed whether a dual suture anchor versus an interference screw fixation is biomechanical superior as a fixation method. They demonstrated that interference screws have a higher ultimate failure load, but suture anchors were able to withstand greater elongation periods [36]. A clinical study reviewed retrospectively patients who had a subpectoral LHB tenodesis with an interference screw or with a suture anchors and hypothesized that the interference screw fixation would offer clinical advantages [37]. In their review of 88 patients, they had no failures with either construct, nor were the VAS pain, or ASES, or Constant scores significantly different, however, there was only one patient in the interference screw group versus four in the suture anchor fixation group that had persistent bicipital groove tenderness [37]. Another study by the same author reported on complications after LHB tenodesis with an interference screw [38]. They were able to include 491 patients with the same tenodesis technique and reported only of 12 cases (2.4%) with complications. One case had a major complication with a proximal humeral fracture at tenodesis site. Eight cases had tendon ruptures, two cases had minor sprains and one screw had to be revised as it was to prominent. They concluded that the subpectoral tenodesis fixation demonstrated excellent clinical outcomes with a low complication rate [38].

Despite our limited clinical data, in our view and with regards to the literature [36–38], a suprapectoral tenodesis site yields inferior clinical results and a subpectoral tenodesis site is preferred to maximize tendon excision, and will result in less revision surgery since there is no damaged tendon left behind.

There are issues associated with the use of the Bonar histopathologic scoring system. First, there are problems linking the Bonar score to clinical and radiological features [39, 40]. Second the original Bonar score assessed only four variables but many authors use a modified five-variable version making comparisons cumbersome [13, 17, 41, 42]. Thirdly, studies using the Bonar score do not always describe the number of investigators used or their level of experience [13, 42]. Finally, investigators usually randomly select an area of the tendon for evaluation limiting the standardization process. One issue for future consideration is the evaluation of tendons using the Bonar score, where therapeutic interventions have been applied to the tendon causing alterations which need to be reflected in a modified Bonar score [39].

This study has limitations. First, it is not a prospective study and second there are no preoperative clinical scores to correlate clinical outcomes and patient improvements after tenodesis. However, the authors believe that this retrospective study with positive pain-response following preoperative image-guided injection eliminates selection bias of LHBT surgery. Second, clinical follow-up is limited to 51%, however the message remains the same particularly regarding the consistent histopathological changes in the entire group and sub-group clinically reviewed. Third, the group sizes for postoperative clinical follow-up were small with 9 and 14 cases for high- and low-grade Bonar groups, respectively, resulting that this study was underpowered for detection of a single SD difference (60.7% power), but the power with 1.5 SDs reaches 91.7% and therefore is good to detect big differences.

## Conclusions

LHBT histopathology following tenodesis with prior 12 months failed conservative treatment demonstrates with the use of the Bonar scoring system chronic degenerative changes with minimal inflammation confirming that the histopathology resembles a tendinosis not a tendinitis. There is no regional localization of histopathological change suggesting removal of subtotal tendon is recommended for complete pain elimination. Interpretation of postoperative clinical outcomes is limited due to a considerable loss of patient follow-up, however, outcomes appear to be independent of histopathologic changes.

## Data Availability

All data has been presented in this manuscript. The data is anonymous. Further data is not available. The data are not publicly available due to data containing information that could compromise research participant privacy/consent.

## Abbreviations

ASES: American Shoulder and Elbow (score)
DASH: Disability of the Arm, Shoulder and Hand
LHB: Long head biceps
LHBT: Long head of biceps tendon
OSS: Oxford Shoulder Score
RC: Rotator cuff
SF-12: Short form survey
SLAP: Superior labral anterior posterior (lesion)
WC: Worker’s compensation
VAS: Visual analogue scale
